# Mapping the Phenotypic Spectrum of Occipital Lobe Epilepsy: A SEEG‐Defined Network Classification

**DOI:** 10.1002/cns.71027

**Published:** 2026-08-07

**Authors:** Changquan Wang, Wenzhe Chen, Jing Hong, Ziyu Mao, Wenzhen Chen, Jiwen Xu

**Affiliations:** ^1^ Department of Functional Neurosurgery, Comprehensive Epilepsy Unit, Ruijin Hospital Luwan Branch Shanghai Jiao Tong University School of Medicine Shanghai China; ^2^ Department of Neurosurgery, Clinical Neuroscience Center, Comprehensive Epilepsy Unit, Ruijin Hospital Shanghai Jiao Tong University School of Medicine Shanghai China

**Keywords:** anatomical–electroclinical classification, clinical phenotype, network dynamics, occipital lobe epilepsy, stereo‐electroencephalography

## Abstract

**Objective:**

Occipital lobe epilepsy (OLE) often presents with complex clinical symptoms that extend beyond the occipital lobe, posing significant challenges for diagnosis and lesion localization. This study aims to systematically characterize the seizure phenotype spectrum of OLE using stereo‐electroencephalography (SEEG) and elucidates its electrophysiological and anatomical basis.

**Methods:**

This single‐center, retrospective case series included 19 patients who underwent comprehensive SEEG evaluation. Seizure phenotypes were classified based on the initial and evolving clinical manifestations. SEEG recordings coregistered with MRI/CT fusion images were used to identify the seizure onset zone and subsequent propagation pathways. Through systematic analysis of electroclinical correlations in conjunction with the dual‐stream model, phenotypic features were mapped to specific propagation networks.

**Results:**

We identified five distinct clinical phenotypes: Pure visual (I), ±visual → automatism (II), visual aura → oculomotor → evolving motor (III), oculomotor onset (IV), and behavioral arrest → motor (V). These mapped to four reproducible SEEG propagation patterns: restricted (I, 10.5%), ventral stream–limbic (II, 26.3%), dorsal‐medial/default mode network–limbic (III, 47.4%), and lateral occipital–temporoparietooccipital junction (TPOJ) (IV, 15.8%). Individual patients could exhibit multiple clinical phenotypes across different seizures. The TPOJ and posterior cingulate/precuneus emerged as key hubs for rapid cross‐network propagation and symptom evolution. Representative cases showed close temporal concordance between clinical semiology and SEEG‐defined propagation sequences.

**Conclusions:**

This study proposes a preliminary anatomical–electrophysiological–clinical classification for OLE. Seizure phenotypes in OLE were associated with structured, pathway‐specific network recruitment rather than nonspecific spread within this single‐center cohort. A phenotype‐driven SEEG approach may offer valuable insights into seizure network organization and could inform presurgical evaluation and individualized surgical planning, though these findings require validation in larger, prospective cohorts.

## Graphical abstract

This SEEG‐based study identifies four propagation patterns in occipital lobe epilepsy (restricted, ventral–limbic, dorsal/DMN–limbic, and lateral TPOJ) linked to five clinical phenotypes. Seizure semiology is shaped by pathway‐selective propagation rather than the onset zone alone, providing a network‐based framework for personalized surgical planning.

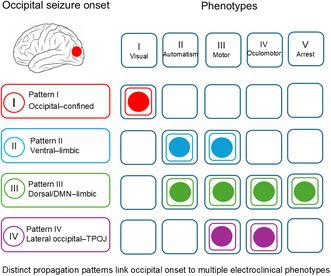

## 1 Introduction

Occipital lobe epilepsy (OLE) involves seizures originating in the occipital lobe [1]. Its extensive connections to other brain regions allow rapid spread of epileptic activity, causing diverse symptoms such as visual auras, visual field defects, hallucinations, forced gaze deviation, nystagmus, and eyelid myoclonus [2, 3, 4]. These often resemble seizures from the temporal or parietal lobes, or the occipitotemporal junction, leading to diagnostic confusion. Scalp electroencephalography (EEG) and standard imaging (particularly in imaging‐negative cases) demonstrate limited efficacy in seizure focus identification, thus substantially increasing risks of misdiagnosis and therapeutic delays [4, 5, 6, 7]. Stereo‐electroencephalography (SEEG) has emerged as a pivotal diagnostic advancement, utilizing depth electrodes to simultaneously capture epileptiform activity from both the occipital cortex and subcortical networks [6, 8]. This technique enables precise identification of seizure foci and adjacent propagation pathways, directly informing individualized surgical or neuro‐modulatory interventions.

Previous studies on the occipital lobe mainly relied on anatomical and symptom analysis, failing to reveal the intrinsic connections among clinical manifestations, structural and electrophysiological features. This gap has limited the understanding of the pathogenesis of occipital lobe epilepsy and hindered the development of personalized treatment. This study analyzed clinical manifestations, neuroimaging, scalp EEG, and SEEG data from patients with occipital lobe epilepsy, integrating the dual‐stream model [9, 10, 11] to build a multidimensional feature analysis system.

## 2 Materials and Methods

### 2.1 Anatomical Definitions and Occipital Lobe Parcellation

The surface anatomy of the occipital lobe is described as irregular and highly complex [12, 13]. In this study, the lateral surface of the occipital lobe includes the superior occipital gyrus (SOG), the middle occipital gyrus (MOG), and the inferior occipital gyrus (IOG). The medial surface includes the parieto‐occipital sulcus (POS) and the calcarine sulcus, which separate the cuneus (Cu), the precuneus (PCu), and the lingual gyrus (LG). The basal surface of the occipital lobe is demarcated anteriorly by an imaginary line extending from the occipital notch to the inferior edge of the splenium of the corpus callosum, thereby separating it from the basal temporal lobe [12].

### 2.2 Study Design and Patient Selection

This single‐center, retrospective case series was conducted at the Epilepsy Center of Ruijin Hospital Luwan Branch, Shanghai Jiao Tong University School of Medicine, from August 2021 to April 2025. The study was approved by the hospital ethics committee, and informed consent was obtained from all patients or their legal guardians. A rigorous multidisciplinary quality control protocol was implemented to ensure data integrity. All datasets underwent independent blinded evaluation by two or more senior neurologists/neuroradiologists specializing in: (1) clinical semiology of epileptic seizures, (2) electrophysiological monitoring (including scalp EEG and SEEG), and (3) neuroimaging modalities: magnetic resonance imaging (MRI) and fluorodeoxyglucose–positron emission tomography (^18^F‐FDG PET). Consensus validation was subsequently achieved through structured multidisciplinary team (MDT) review sessions. Inclusion criteria: (1) preoperative noninvasive evaluation showing diagnostic features of occipital lobe epilepsy, including characteristic seizures and neuroimaging or EEG findings; (2) SEEG‐confirmed seizure onset in the occipital lobe; (3) complete clinical records and available follow‐up data. Exclusion criteria: (1) atypical seizure onset; (2) severe systemic comorbidities; (3) incomplete data or loss to follow‐up.

### 2.3 Clinical Multimodal Assessment

#### 2.3.1 Clinical Data Collection

A standardized clinical assessment protocol was implemented for systematic data collection. Patient demographics, epilepsy history, imaging results, and surgical data were collected via the electronic medical record system. Postoperative follow‐up assessed seizure outcomes using the Engel classification.

#### 2.3.2 Neuroimaging Acquisition

The MR study included T1 magnetization‐prepared rapid acquisition gradient echo (MPRAGE) (voxel size 0.5 × 0.5 × 0.5 mm), T2 sequences (voxel size 0.3 × 0.5 × 0.3 mm), T2‐Flari sequences (voxel size 0.5 × 0.5 × 0.5 mm), and phase contrast magnetic resonance angiography (voxel size 0.37 × 0.37 × 0.37 mm) using a 3.0‐T scanner (uMR 890; United Imaging Healthcare, Shanghai, China) with a dedicated 64‐channel head coil. Patients were required to fast and abstain from sugary beverages for a minimum of 6 h prior to the procedure. Following intravenous administration of ^18^F‐FDG (0.1 mCi/kg), subjects were instructed to rest in a quiet, dimly lit environment for 30 min before proceeding to standardized imaging protocol acquisition.

#### 2.3.3 Scalp Electroencephalography

All patients underwent long‐term video scalp EEG monitoring. Electrodes were placed according to the international 10–20 system using a 32‐channel configuration. Recording parameters were set as follows: a sampling rate of 1024 Hz, a high‐pass filter at 0.5 Hz, and a low‐pass filter at 70 Hz. Both interictal and ictal changes were assessed.

### 2.4 SEEG Recording and Analysis

#### 2.4.1 Electrode Implantation Strategy and Recording Parameters

SEEG implantation plan is based on noninvasive assessment results, including clinical manifestations, MRI, PET, and neuropsychological assessment. The individualized electrode placement is formulated by the MDT of the epilepsy center and precisely covers the key subregions of the occipital lobe and potential transmission pathways. SEEG recordings were acquired using the Nihon Kohden system, recording activity from 256 contacts at 1024 Hz with a 0.05–600 Hz bandpass filter. Drawing on established methodologies, six distinct SEEG seizure onset patterns were characterized [14]. Systematic evaluation of ictal activity characteristics and temporal dynamics across electrode contacts enabled precise determination of discharge propagation sequences, with subsequent reconstruction of electro‐anatomical propagation pathways.

#### 2.4.2 Antiseizure Medication Management During Monitoring

Baseline ASM doses were maintained for 5 days. Patients with frequent spontaneous seizures underwent no tapering. For others, ASMs were tapered stepwise, one at a time, by 25%–50% every 24 h, prioritizing recently added agents; benzodiazepines and barbiturates were reduced most cautiously due to withdrawal risk. All adjustments were supervised by a senior epileptologist, and doses were promptly restored or modified in the event of seizure clustering, prolonged seizures, or intolerance. Final propagation‐pattern classification relied on habitual seizures with concordant electroclinical features; seizures showing atypical features only after substantial ASM reduction were interpreted cautiously and excluded from primary classification.

#### 2.4.3 Assessment of Electrode Coverage Adequacy and Propagation‐Pattern Assignment

For propagation‐pattern analysis, we predefined a set of downstream pathway‐specific nodes based on the dual‐stream organization of occipital visual networks and prior anatomo–electroclinical evidence (Table S1). These nodes included representative relay regions within the ventral, dorsal, and posterior midline systems that were considered most relevant to electroclinical propagation‐pattern classification. Electrode coverage for each key node was assessed using postimplantation CT–MRI fusion. Coverage was classified as fully covered (≥ 2 gray‐matter contacts), partially covered (1 gray‐matter contact or suboptimal sampling), or not covered (no contact within the ROI). Because SEEG implantation was hypothesis‐driven and guided by Phase I presurgical evaluation, coverage varied across regions.

Pattern assignment relied on the predominant electroclinical pathway from SEEG temporal dynamics and ictal recruitment anatomy. Complete sampling of all nodes was not required; patterns were assigned when minimally sufficient nodes were sampled. To minimize misclassification, a pathway component was included only if its defining downstream key nodes were directly recorded. When key nodes were incompletely sampled, preoperative FDG‐PET, scalp EEG, and semiology provided supportive evidence. In such cases, pathway inference was additionally supported if at least two modalities concurred with the SEEG pattern. Final classification used habitual seizures with concordant electroclinical features to identify the predominant propagation stream, not an exclusive anatomical route. The criteria for coverage assessment and multimodal validation are summarized in Table S1.

### 2.5 Phenotype Adjudication and Representative Case Selection

Two senior epileptologists blinded to each other's SEEG findings independently reviewed all seizures, classified semiology using video‐EEG/clinical descriptions and SEEG‐anchored temporal evolution, and assigned phenotype labels. Disagreements were resolved by joint review of seizure segments and clinical notes; unresolved cases were adjudicated by a third epileptologist to reach consensus. Representative cases were selected based on: (1) seizures with canonical semiologic evolution for the phenotype; (2) artifact‐free SEEG recordings showing clear ictal onset and propagation; (3) comprehensive electrode coverage of relevant propagation pathways; and (4) habitual seizures confirmed by caregiver report or prior video‐EEG. When multiple seizures met all criteria, the most electrographic and clinical features were selected.

## 3 Results

### 3.1 Clinical Characteristics

Between 2021 and 2025, 19 patients were diagnosed with occipital lobe epilepsy through SEEG. The cohort included 10 males and 9 females, with a mean age of 19.7 ± 7.3 years and a mean age at seizure onset of 11.7 ± 6.8 years. The epileptogenic zone was lateralized to the left hemisphere in 11 patients (57.9%) and to the right hemisphere in eight patients (42.1%). Neurocognitive function, evaluated using either the Wechsler Adult Intelligence Scale or the Wechsler Intelligence Scale for Children, was available for 17 patients, revealing Intelligence Quotient scores ranging from 60 to 111. Two patients were unable to complete the neuropsychological assessment (Table 1).

**TABLE 1:** Scalp EEG, imaging findings, cognitive function, surgical intervention, and postoperative pathology with occipital‐onset epilepsy.

Pat. no.	Sex	Age (years)	Age at onset (years)	Lat.	Preop MRI	Scalp‐EEG		Preop FDG‐PET hypometabolism	WAIS/WISC	Surgical treatment	Postop. follow‐up (months)	Postop. path.	Engel
						IID	ID						
1	F	23	13	L	Cu‐POS FCD	Bil. Pos. Bil. Ant.	Bil. Pos. L > R	L Cu, POS, PCu, HIP	95	RFTC and resection	43	FCD IIb	IA
2	F	11	9	R	MOG Gliosis	R Oc R T	R Oc R T	R Oc, P, T	94	RFTC	43	No	IA
3	F	23	16	L	Normal	L Oc	L Oc	L LG, IOG	100	RFTC and resection	37	FCD IIa	IC
4	F	27	23	L	MOG‐PCC FCD	Bil. T	Bil. T L > R	L MOG, PCC, SOG. B. (L > R) T, HIP	78	RFTC	31	No	IB
5	F	26	15	R	SOG‐Cu FCD	R Pos. Bil. T	R Pos.	R Cu, T, HIP	90	RFTC and resection	31	FCD IIb	IIA
6	M	21	14	L	Boc lesion	L Oc L T	L Oc L T	L LG, FuG, IOG, T, HIP, PHG	95	RFTC and resection	30	GG	IA
7	M	26	11	R	Bil. Pos. Gliosis	Bil. Pos.	Bil. Pos. R > L	B. (R > L) Oc, HIP R SPL, Cu	60	RFTC	30	NO	III
8	M	17	15	R	MOG FCD	Bil. Pos. R > L	Bil. Oc	R MOG, SPL, HIP	67	RFTC and resection	30	FCD IIb	IA
9	F	14	3	L	IOG‐BOc Gliosis	L Oc L T	L Oc L T	L MOG, IOG, T, PHG, HIP	95	RFTC	29	No	IA
10	M	15	9	L	Bil. Cu Gliosis	L Oc L P	L Oc	B. Cu	60	RFTC	25	No	IA
11	M	40	28	L	Bil. Pos. encephalomalacia	Bil. Oc Bil. T	Bil. Oc R > L	B. (R > L) Pos, T	—	RFTC and resection	25	Gliosis	IB
12	M	13	2	R	Normal	Bil. Oc&T R > L	R Oc R T	R Oc, T, HIP	—	RFTC	19	No	III
13	F	17	14	L	Normal	Bil. O L T	L Oc L T	L Oc, T, FuG, HIP	111	RFTC	19	No	IA
14	F	17	2	L	Normal	Bil. Pos L > R	L Oc, L T R Oc, R T	L IOG, MOG, ITG, MTG, B. HIP	102	RFTC	17	No	III
15	M	24	16	R	LG FCD	R Oc R T	R Oc R T	L LG, Cu, FuG B. (R > L) HIP	107	RFTC and resection	7	FCD IIb	IB
16	F	9	2	L	L Oc encephalomalacia	Bil. Oc, L P, Bil. T	L Oc, L P, L T	L MOG, IPG, STG, MTG, HIP	84	RFTC	5	No	II
17	M	12	6	L	Bil. Pos. encephalomalacia	L Oc L T	L Oc L T	L Cu, PCu, SOG, IOG, SPL, PCC, T, HIP	78	RFTC	5	No	II
18	M	25	14	R	Cu Gliosis	L Ant Bil. Pos	Bil. Pos R > L	R Cu, PCu, SPL	96	RFTC	5	No	IA
19	M	15	11	R	Bil. Pos. Gliosis	Bil. Pos R > L	Bil. Pos R > L	R Cu, SOG, MOG B. HIP	76	RFTC	4	No	II

Abbreviations: Ant, anterior; Bil, bilateral; Cu, cuneus; F, female; FCD, focal cortical dysplasia; GG, ganglioglioma; ID, ictal discharge; IID, interictal discharge; L, left; Lat, lateralization; M, male; Oc, occipital cortex; Pat. no., patient number; Path, pathology; Pos, posterior; R, right; resection, surgical resection of the epileptogenic zone; RFTC, SEEG‐guide radiofrequency thermocoagulation; T, temporal; WAIS, Wechsler Adult Intelligence Scale; WISC, Wechsler Intelligence Scale for Children.

### 3.2 Neuroimaging Findings

In this group of cases, there were four negative patients, five cases of focal cortical dysplasia (FCD), six cases of gliosis, three cases of encephalomalacia, and one case of tumor. Despite potential false‐positive findings and inconsistent tabular reporting, available data indicate that hypometabolic areas identified by FDG‐PET correlate with structural and electrophysiological findings, frequently delineating affected occipital regions and their propagation pathways to temporal or parietal areas.

### 3.3 Scalp EEG Findings

Scalp EEG showed heterogeneous findings with predominant posterior quadrant abnormalities, consistent with an occipital lobe origin. Interictal epileptiform discharges (IEDs) appeared in posterior regions, occipital, parietal, or temporal, and were often unilateral, though bilateral discharges with asymmetric predominance occurred frequently. Ictal onset was mainly in the occipital, parietal, or temporal areas. Some patients had bilateral onset, and many seizures spread rapidly to the temporal lobe, indicating fast propagation from occipital regions.

### 3.4 SEEG Analysis

SEEG electrodes were unilaterally implanted in 15 patients (76.5%) and bilaterally in four patients (23.5%), with a mean of 11.2 ± 2.2 electrodes per patient. SEEG implantation employed orthogonal and oblique trajectories to ensure comprehensive coverage of the occipital lobe, including its lateral, basal, and medial surfaces, with precise targeting of structures such as the LG, Cu, fusiform gyrus (FuG), and lateral occipital cortex. Based on individualized presurgical hypotheses, additional key regions were implanted, encompassing the cingulate gyrus, PCu, para‐hippocampal gyrus (PHG), amygdalohippocampal complex, temporo‐parieto‐occipital junction (TPOJ), superior parietal lobule (SPL), inferior parietal lobule (IPL), and temporal neocortex. This approach effectively targeted the primary visual cortex, downstream visual networks (dorsal and ventral streams), as well as limbic, oculomotor, and multimodal association hubs (Table 2).

**TABLE 2:** Seizure onset zone, SEEG propagation patterns, and per‐seizure phenotype–pattern assignments.

Pat. no.	Sz symptoms in order of emergence	SEEG side	n‐electrode	Electrode implantation trajectory (entry point‐target point)	SOZ (SEEG propagation)	SOP	Pattern	Phenotype
1	Sz1. Motor arrest (staring) → tonic posturing of the left upper limb → head and eye deviation to the left → sGTCS	L‐uni	12	PostCG‐PCC, SPL‐PCC, SPL‐PCC, Oc‐Cu, Oc‐LG*2, Oc‐PCu*2, MTG‐PCC, MTG‐body‐HIP, ITG‐AMG, MTG‐head‐HIP	Lesion (Cu‐POS) > PCu > PCC > HIP	LVFA	III	V
	Sz2. Staring → oropharyngeal automatisms → eye blinking → staring with unresponsiveness.				Lesion (Cu‐POS) > PHG > POS > HIP		III	II
2	Eyes L‐deviation → asymmetric tonic posturing → vocalization → eye blinking → head/neck hyperextension → sGTCS.	R‐uni	13	Oc‐PCu, Oc‐PCC, SPL‐Lesion cortex, SMG‐pINS, TPOJ‐Isth, TPOJ‐Isth, Lesional Cortex‐CoS, TPOJ‐PCC, pMTG‐LG, TPOJ‐Isth, MTG‐Tail of HIP, SMG‐TP, STG‐INS	Lesion (MOG) > TPOJ > SPL	Preictal rhythmic spikes	IV	IV
3	Aura: Elementary visual hallucinations (black/white spots → enlarging → colored spots) → headache	L‐uni	9	SOG‐LG*3, MOG‐Cu*3, IOG‐LG*3	LG > IOG	LVFA	I	I
4	Motor arrest, behavioral pause (→ tachycardia ~30%) → eyes L‐deviation → bilateral (R > L) or R upper limb tonic posturing and bilateral mouth deviation (downwards) → sGTCS	L‐uni	13	SPL‐PCC *3, Oc‐Isth, Oc‐LG, Oc‐PCu, Oc‐Cu*3, MTG‐PCC, STG‐ALG, STG‐PSG, MTG‐head of HIP	Lesion (MOG) > PCC > PCu > HIP	Preictal rhythmic spikes	III	V
5	Motor arrest, behavioral pause; tachycardia (~80%) → R hand automatisms and eye blinking and head/eye/body deviation to R → L upper limb tonic posturing → bilateral hand automatisms	R‐uni	9	SPL‐PCC, SPL‐Isth, Oc‐Cu*2, Oc‐LG*2, Oc‐FuG, Oc‐PCu, MTG‐FuG	Lesion (SOG) > SPL > PCC > MOG	LVFA	III	V
6	Sz1. Aura: Elementary visual hallucinations (yellow‐green spots in R visual field) → may progress to: repetitive head/eye pursuit to R → oroalimentary automatisms and L hand automatisms.	L‐uni	8	SOG‐LG*2, MOG‐LG*2, IOG‐LG, IOG‐FuG*2, ITG‐FuG	Lesion (LG) > FuG > ITG	Preictal rhythmic spikes	II	II
	Sz2. Aura: Elementary visual hallucinations → head and eye deviation to L → sGTCS				Lesion (LG) > FuG > ITG		II	III
7	Sz1. Aura: visual loss (amaurosis) → orolimentary automatisms and eye blinking.	Bil.	9	R. SPL‐Isth, Oc‐Cu*2, Oc‐LG*2, SPL‐PCC L. Oc‐Cu, Oc‐LG*2	Lesion (Cu) > PCu > PCC	LVFA	III	II
	Sz2. Motor arrest → head and eye deviation to L → asymmetric tonic posturing → vocalization → bilateral clonic ending with R‐sided clonic.				Lesion (Cu) > PCu > PCC > SOG > SPL		III	V
8	Aura: Visual obscuration → motor arrest (object dropping) → eyes L‐pursuit → R hand automatisms and L upper limb tonic posturing.	R‐uni	12	SPL‐POS, SPL‐CalS, Oc‐LG, SMG‐Isth, TOJ‐CalS, Oc‐Cu, Ang‐LG, AngG‐PCC, MTG‐LG, MTG‐head of HIP, SPL‐LI	Lesion (MOG) > TPOJ > Cu > PCu > MTG	LVFA	IV	III
9	Eye blinking → eyes R‐pursuit → L hand automatisms and R upper limb tonic posturing → (May progress to) R mouth deviation → bilateral asymmetric tonic posturing.	L‐uni	13	SPL‐Isth, Oc‐LG, SPL‐PCu, Oc‐Cu, Oc‐LG, TPOJ‐Cu*2, ITG‐PHG, MTG‐PHG*2, SMG‐PCC, MTG‐AMG, MT‐head of HIP	Lesion (MOG) > MTG > TPOJ > SMG	Preictal rhythmic spikes	IV	IV
10	Aura: Elementary visual hallucinations (white flashes) → Headche	Bil.	11	L. SOG‐PCu, SOG‐LG*3, MOG‐Cu*2, IOG‐LG*3 R. MOG‐LG, SOG‐LG	Lesion LG > Cu	Preictal rhythmic spikes	I	I
11	Orolimentary automatisms (chewing/swallowing) → R upper limb automatisms → vocalization/verbal automatisms.	L‐uni	7	Oc‐LG, Oc‐FuG, Oc‐Cu, MTG‐head of HIP, MTG‐FuG*2, ITG‐FuG	Lesion (IOG) > ITG > FuG > HIP	Preictal rhythmic spikes	II	II
12	Aura: Visual loss (amaurosis) → eyes L‐pursuit → eye blinking → orolimentary automatisms.	R‐uni	12	SOG‐Cu*2, Oc‐LG*4, MTG‐AMG, MTG‐HIP (body), MTG‐tail of HIP, MTG‐head of HIP, AngG‐PCC, SPL‐PCC	LG > FuG > PHG > HIP	LVFA	II	II
13	Aura: Dizziness (+/− colored lights) → minor variant: eye blinking; major variant: head/eye deviation to R → bilateral asymmetric tonic posturing → sGTCS	L‐uni	14	SPL‐rspCtx, Oc‐LG*3, AngG‐LG, SMG‐PCC, AngG‐LG, ITG‐PHG, ITG‐FuG, MTG‐head of HIP, SPL‐PCC, TPOJ‐PCC, MTG‐LG, AngG‐PCu.	IOG > ITG > MOG > HIP	LVFA	II	III
14	Aura: visual obscuration → head and eye deviation to R → sGTCS.	Bil.	14	L. SPL‐PCu, SOG‐LG*2, AngG‐PCC, AngG‐Cu, IOG‐LG, STG‐WM, MTG‐rspCtx, ITG‐PHG*2, MTG‐HIP R. SOG‐LG, SOG‐LG, AngG‐PCC	LG > FuG > IOG > ITG	Rhythmic spike–wave	II	III
15	Sz1: Orolimentary automatisms → (chewing, swallowing) → eye blinking	R‐uni	13	SOG‐LG*2, IOG‐LG*3, MOG‐Cu, MOG‐LG, MOG‐LG, MTG‐HIP*2, ITG‐Tail of HIP, MOG‐PCu	Lesion (LG) > Cu > HIP > PCu	Preictal rhythmic spikes	III	II
	Sz2: Sz1 → progress to head and eye deviation to L → sGTCS.				Lesion (LG) > Cu > HIP > PCu		III	II
16	Motor arrest → eye blinking → head and eye version to the right.	L‐uni	10	SOG‐LG, IOG‐LG, PostCG‐MCC, SPL‐PCC, PreCG‐Pre‐lesion, PreCG‐Intra‐lesion, MOG‐PCu, AngG‐PCu, IOG‐PCu, MTG‐HIP	Lesion (SOG) > SPL > PCu > PCC > MCC > HIP	Preictal rhythmic spikes	III	V
17	Aura: Visual obscuration (+/− spatial distortion) → eyes R‐pursuit and eye blinking → head and neck version to R → R‐sided clonic (mouth, face, neck, upper limb).	L‐uni	8	SOG‐LG, LG‐PHG, SOG‐PCC, IOG‐PHG, MOG‐PHG, Ang‐PCC, SPL‐PCC, IPL‐PCC	Lesion (LG) > FuG > PCu > SPL	LVFA	III	III
18	Eye blinking → asymmetric facial movements (R eye closure and L eye blinking) → orolimentary automatisms and R hand automatisms → head and eye deviation to L → sGTCS.	R‐uni	13	MTG‐AMG, SPL‐PCC*3, SOG‐rspPCC, SOG‐LG, IPL‐PHG, IPL‐LG*2, IPL‐MCC, MTG‐ACC, Oc—pINS, MTG‐head of HIP	Lesion (Cu) > PCu > PCC > HIP	LVFA	III	IV
19	Aura: Elementary visual hallucinations (colored lights) → eye blinking → eyes/head deviation to L → evolving to focal clonic jerks (head).	Bil.	13	R. SPL‐PCC, SOG‐LG, SMG‐PCC, AngG‐PCu, AngG‐PCC, Oc‐LG*2, Oc‐FuG, MTG‐LG, MTG‐tail of HIP, MTG‐body of HIP L. SPL‐PCu, SOG‐LG	Lesion (MOG) > PCu > PCC > HIP > MTG	LVFA	III	III

Abbreviations: ACC, anterior cingulate cortex; AMG, amygdala; AngG, angular gyrus; aSMG, anterior supramarginal gyrus; Bil, bilateral; BOc, basal occipital; CalS, calcarine sulcus; CoS, collateral sulcus; Cu, cuneus; FuG, fusiform gyrus; HIP, hippocampus; IOG, inferior occipital gyrus; IPS, intraparietal sulcus; Isth, isthmus of cingulate gyrus; ITG, inferior temporal gyrus; Lat‐Oc, lateral occipital cortex; LG, lingual gyrus; LVFA, low‐voltage fast activity; mBody, mid‐body; MCC, middle cingulate cortex; MTG, middle temporal gyrus; Oc, occipital lobe; Pat. no., patient number; PCC, posterior cingulate cortex; PCu, precuneus; PHG, para‐hippocampal gyrus; pINS (PLG), posterior insula (posterior long gyrus); POJ, parieto‐occipital junction; POS, parieto‐occipital sulcus; PostCG, postcentral gyrus; PreCG, precentral gyrus; pSTG, posterior superior temporal gyrus; rspPCC, retro‐splenial part of PCC; SMG, supramarginal gyrus; SOG, superior occipital gyrus; SPL, superior parietal lobule; sup., superior to; TOJ, temporo‐occipital junction; TP, temporal plane; TPO, temporo‐parieto‐occipital junction; WM, white matter.

#### 3.4.1 Seizure‐Onset Zone Localization

Seizure‐onset zones demonstrated a clustered distribution, primarily localized to the primary visual cortex and adjacent occipital regions, with additional involvement of the occipitotemporal and parieto‐occipital border zones. Specifically, the seizure‐onset regions were predominantly identified in the medial occipital cortex in seven cases, the basal occipital area in three cases, and the lateral occipital cortex in nine cases. Low‐voltage fast activity (LVFA) and pre‐ictal rhythmic spikes were the most frequent seizure‐onset patterns.

#### 3.4.2 Ictal Semiology and Seizure Capture During SEEG Monitoring

**FIGURE 1:**
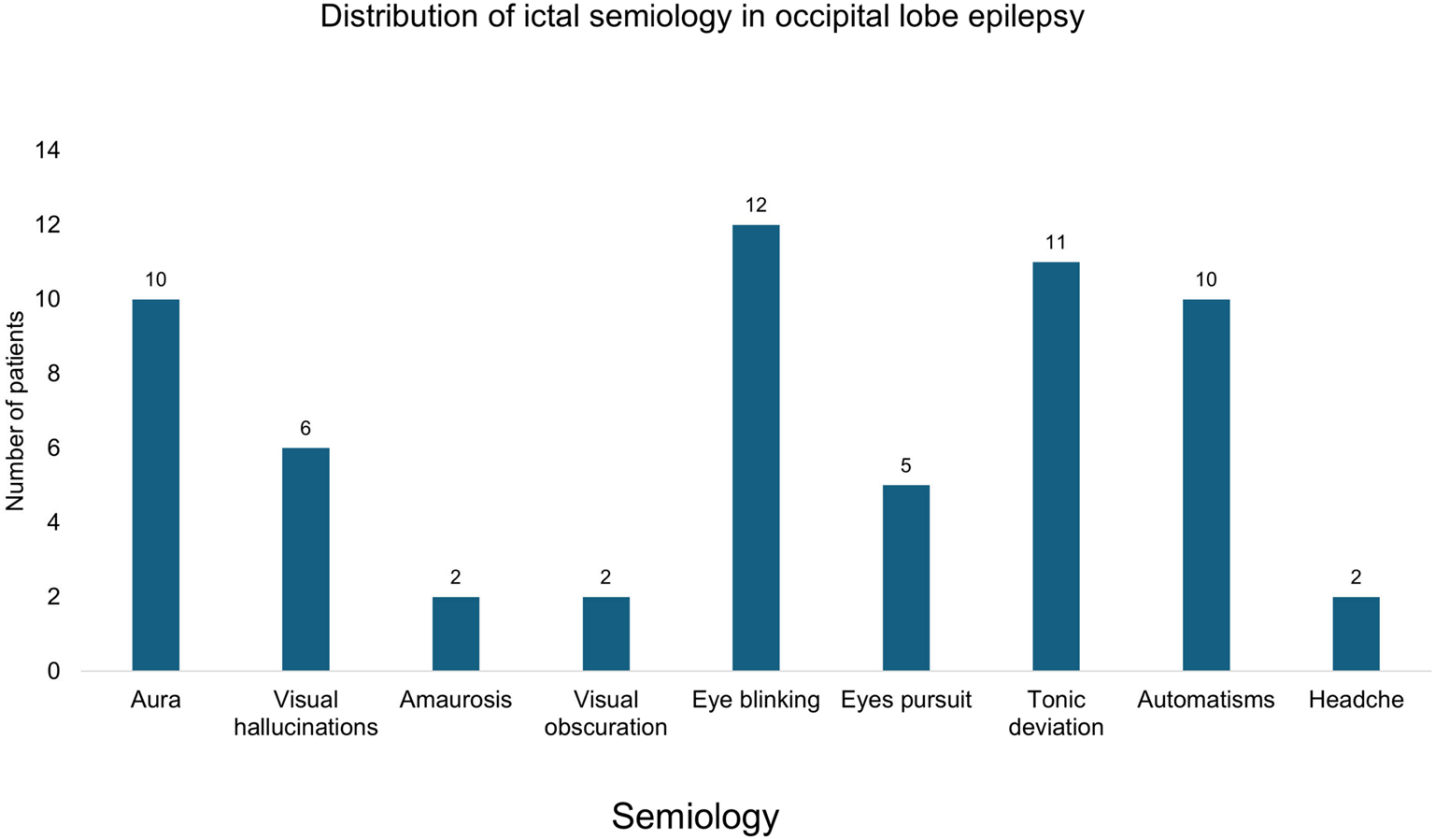
Distribution of ictal semiology in occipital lobe epilepsy. The bar chart illustrates the number of patients presenting with visual, oculomotor, automatism, and motor‐related ictal symptoms.

During SEEG monitoring, each patient experienced three or more clinically documented seizures. Ictal semiology included auras in 10 patients (52.6%), with elementary visual hallucinations in six (31.6%) and amaurosis or visual obscuration in four each (21.1%). Eye blinking occurred in 12 patients (63.2%), tonic eye deviation in 11 (57.9%), and orolingual/hand automatisms in 10 (52.6%). Additional features included eye pursuit in five patients (26.3%) and headache in two (10.5%). This symptom constellation, dominated by visual auras, frequent eye blinking, and oculomotor manifestations, is consistent with occipital lobe seizure onset and aligns with the established propagation patterns of the occipital epileptic network (Figure 1). ASM tapering was implemented in 17 of 19 patients, increasing seizure captures from 41 to 98. Semiology remained fully concordant with habitual events in 17 patients; in the other 2, 1–2 atypical seizures (with contralateral version) occurred but were excluded from propagation‐pattern classification because they deviated from habitual semiology and reflected inadequate contralateral electrode coverage. Thus, tapering improved yield without affecting final classification. Details are provided in Table S2.

#### 3.4.3 Clinical Phenotypes Based on Ictal Semiology Evolution

Based on the sequential progression of ictal semiological features (Table 2), five distinct clinical phenotypes were characterized.

Phenotype I: Pure visual phenotype—This subtype is defined by the exclusive occurrence of subjective visual auras, including elementary visual hallucinations, amaurosis, or visual obscuration, without progression to objective motor or autonomic manifestations. Cases 3 and 10 represent this phenotype.

Phenotype II: ±Visual aura → automatism Phenotype—This subtype involves seizures primarily characterized by automatisms (oropharyngeal, limb, or verbal), with or without a preceding visual aura. Cases 1 (Sz2), 6 (Sz1), 7 (Sz1), 11, 12, and 15 represent this phenotype.

Phenotype III: Visual aura → oculomotor → evolving motor phenotype—Following a visual aura, this form rapidly progresses to head‐eye deviation or eye pursuit, followed by contralateral limb tonic posturing or asymmetric tonic posturing, with some cases evolving to GTCS. Cases 6 (Sz2), 8, 13, 14, 17, and 19 represent this phenotype.

Phenotype IV: Oculomotor‐onset → evolving motor phenotype (without clear visual aura)—Seizures begin with eye blinking or eye pursuit, followed by oral/hand automatisms and contralateral upper limb tonic or asymmetric posturing, which may progress to GTCS. Representative cases include 2, 9, and 18.

Phenotype V: Behavioral arrest onset → oculomotor/motor phenotype—Characterized by initial behavioral arrest (sustained cessation of ongoing voluntary behavior, including speech and limb movement, with reduced responsiveness lasting ≥ 3 s on synchronized video‐EEG/SEEG review), followed by oculomotor signs and limb tonic or hypermotor activity, with a tendency to evolve into GTCS. Representative cases include cases 1 (Sz1), 4, 5, 7 (Sz2), and 16.

#### 3.4.4 Classification of Ictal Propagation (Anatomical–Electrical Pattern)

**FIGURE 2:**
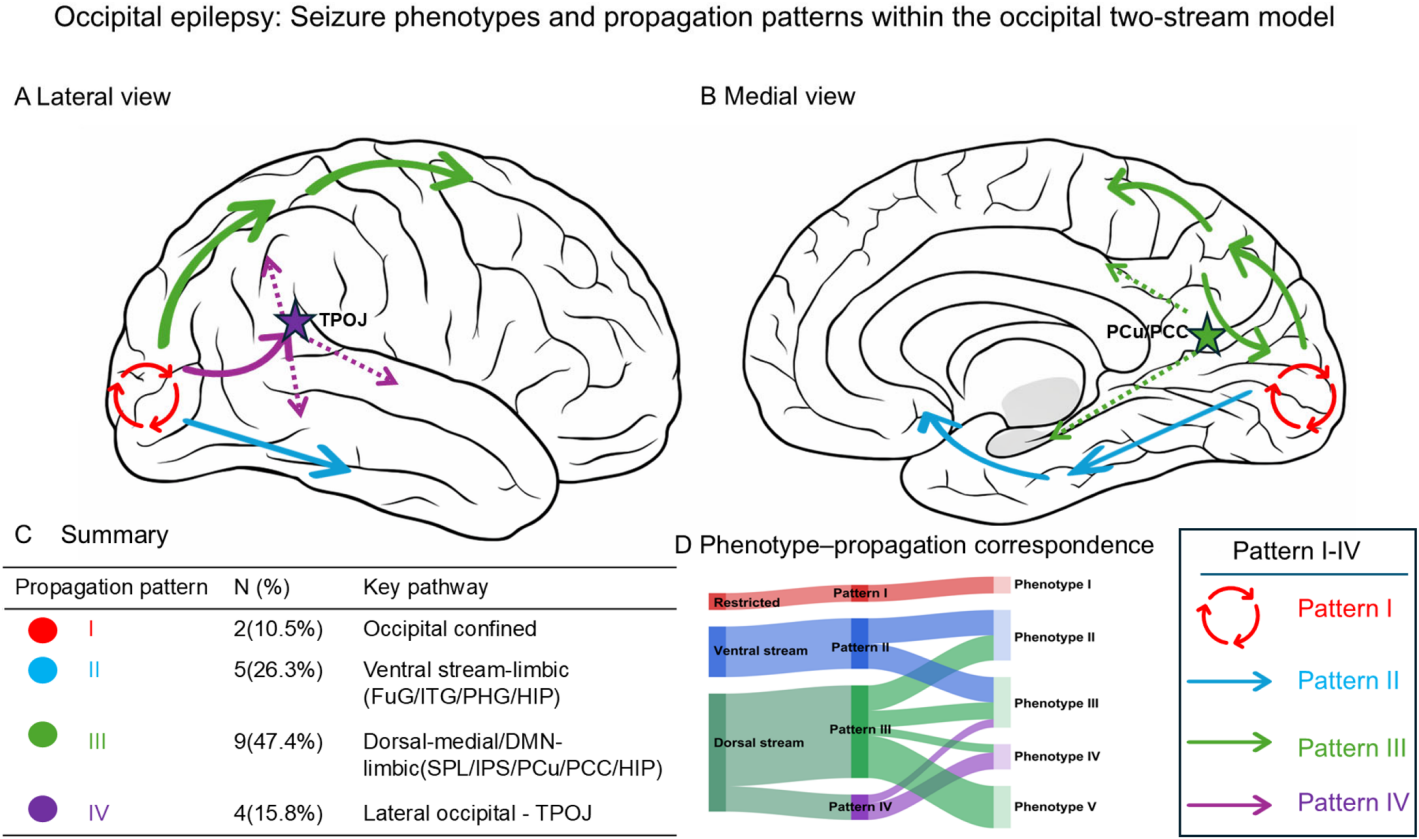
Occipital epilepsy: Seizure phenotypes and propagation patterns within the occipital two‐stream model. (A) Lateral view showing four propagation patterns originating in the occipital cortex: Pattern I (red circular arrow; occipital‐confined), Pattern II (blue arrow; ventral stream–limbic propagation via fusiform and inferior temporal regions), Pattern III (green arrows; dorsal‐medial/DMN–limbic propagation), and Pattern IV (purple arrow; lateral occipital spread toward the temporo‐parieto‐occipital junction, TPOJ; dotted segments indicate indirect routes). (B) Medial view showing Pattern III propagation along the dorsal‐medial/DMN–limbic pathway, involving medial parietal and limbic structures (PCu/PCC, HIP). (C) Summary table reporting, for each pattern (I–IV), the number of cases (*N*, %), key pathway. (D) Sankey‐style flow diagram summarizing the correspondence between propagation patterns and major clinical seizure phenotypes. The diagram shows that individual propagation patterns may be associated with multiple phenotypes, and vice versa. Colors match panels A–C; band width reflects the number of patients per pattern–phenotype pairing, offering an intuitive quantitative overview. Color code: red = Pattern I, blue = Pattern II, green = Pattern III, purple = Pattern IV. Cu, cuneus; DMN, default mode network; FuG, fusiform gyrus; HIP, hippocampus; IPS, intraparietal sulcus; ITG, inferior temporal gyrus; PCC, posterior cingulate cortex; PCU, precuneus; PHG, para‐hippocampal gyrus; SPL, superior parietal lobule; TPOJ, temporo‐parieto‐occipital junction.

Given the complexity of epileptic seizures and the spatial coverage limitations of intracranial electrode sampling, this study systematically classified the epileptic propagation patterns into four types by integrating seizure symptomatology, seizure‐onset pattern and the occipital two‐stream model (Figure 2). The most common propagation pattern involves rapid activation of the dorsal stream (Cu, PCu, SPL, IPS, and/or posterior cingulate cortex [PCC]), with some patients showing near‐synchronous or early involvement of ventral stream structures and limbic structures (FuG, ITG, MTG, HIP). This dual‐stream convergence is typically mediated by key bridging hubs when sampled, especially the TPOJ and the PCC/PCu.

Pattern I (2 patients, cases 3 and 10, 10.5%): Restricted propagation. Epileptic activity remains confined to the occipital lobe, with no consistent extra‐occipital spread. Seizures present pure visual auras, without objective motor or autonomic symptoms.

**FIGURE 3:**
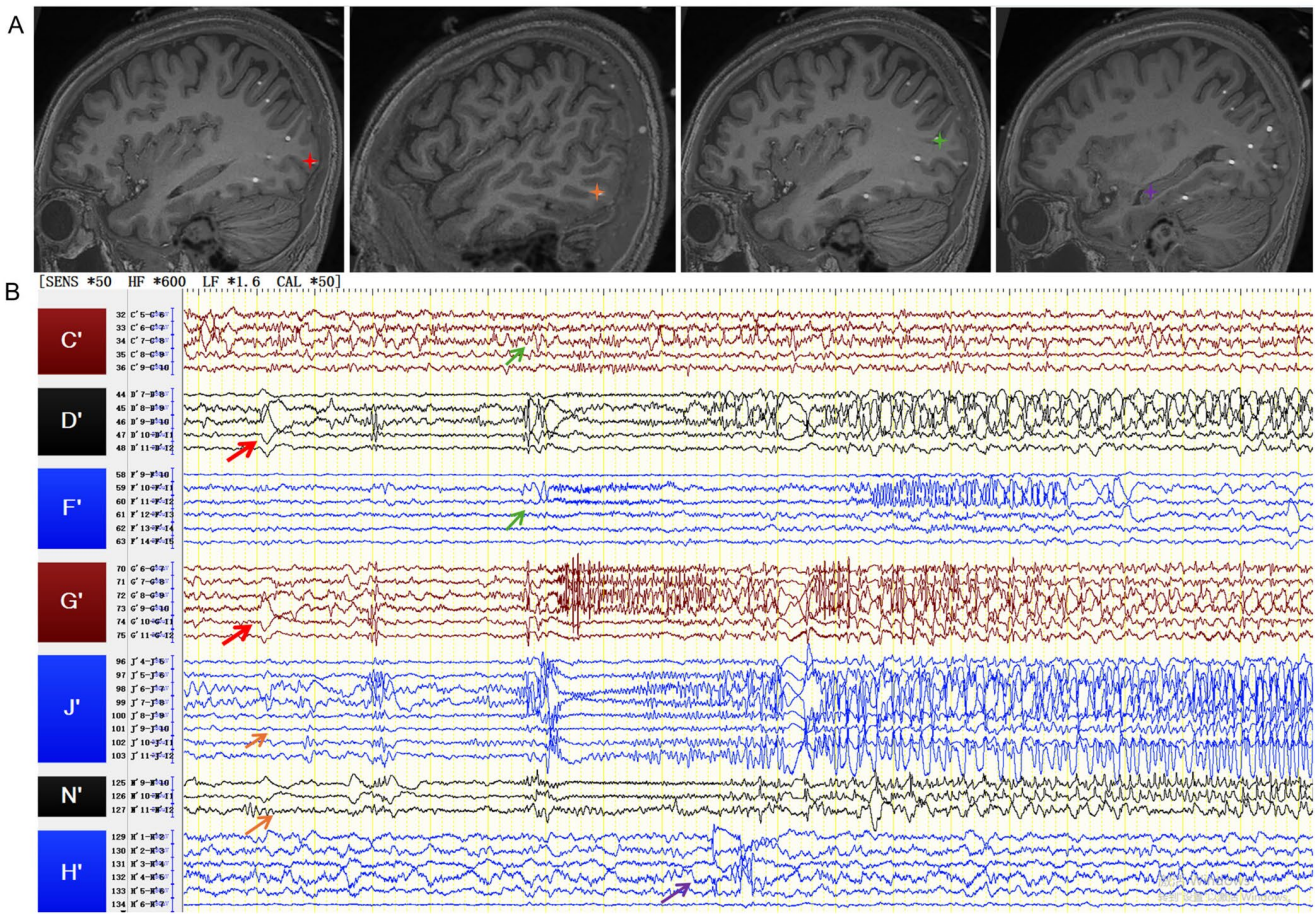
Case 14: SEEG electroclinical correlations. (A) Anatomical localization of key electrode contacts based on MRI/CT fusion imaging. (B) SEEG‐defined electro‐anatomical propagation pattern: IOG (red arrows) → ITG (orange arrow) → MOG (green arrow) → HIP (purple arrow). Electrode contact colors in A match the arrow colors in B. HIP, hippocampus; IOG, inferior occipital gyrus; ITG, inferior temporal gyrus; MOG, middle occipital gyrus.

Pattern II (5 patients, cases 6 and 11–14, 26.3%): Ventral stream–limbic pathway. Activity spreads selectively along the ventral visual stream from the occipital lobe (LG/IOG) to ventral temporal regions (FuG and ITG), with consistent involvement of medial temporal limbic structures, including PHG and HIP (Figure 3).

**FIGURE 4:**
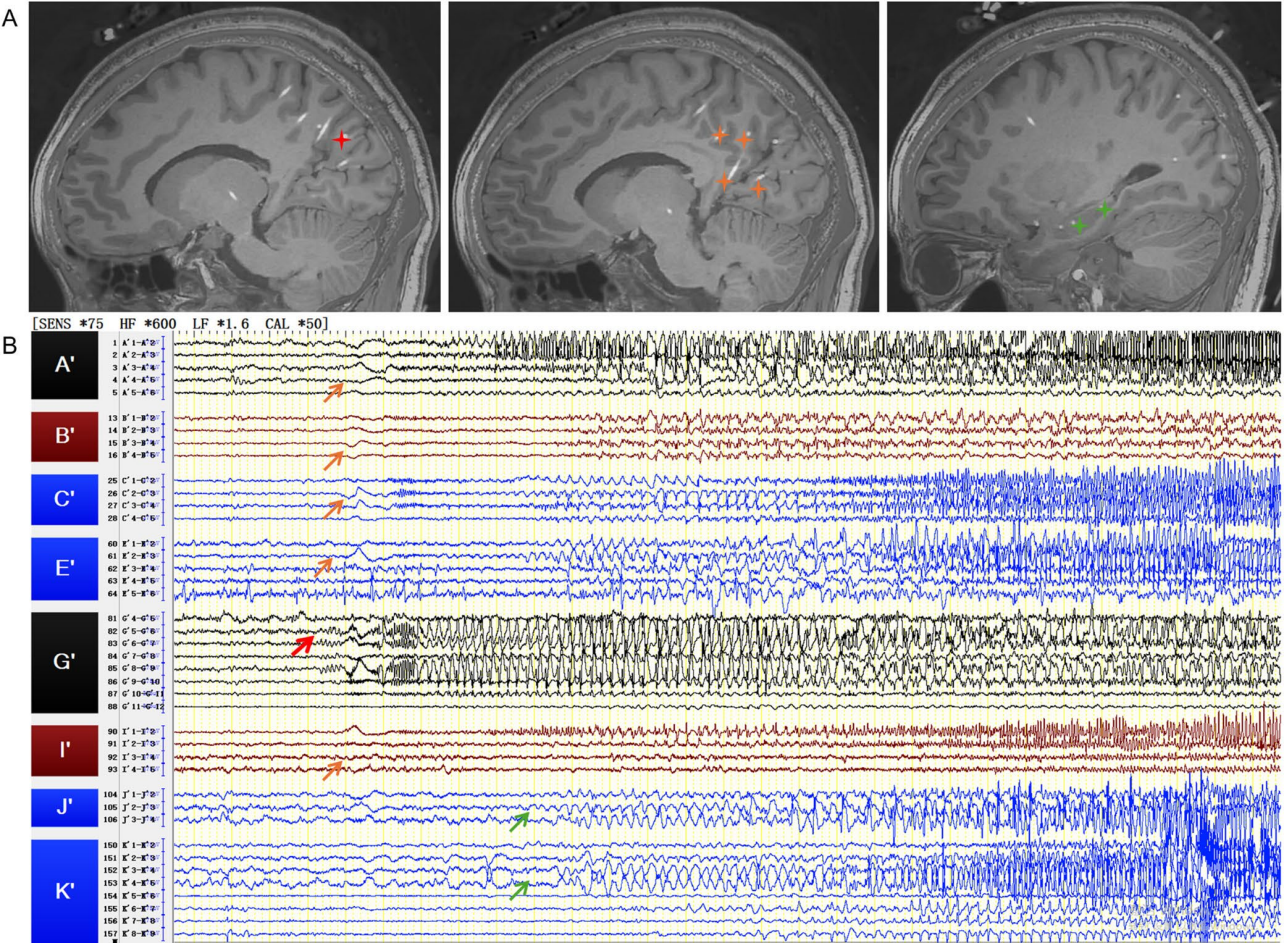
Case 1: SEEG electroclinical correlations. (A) Anatomical localization of key electrode contacts based on MRI/CT fusion imaging. (B) SEEG‐defined electro‐anatomical propagation pattern: Cu (red arrows) → PCu‐PCC (orange arrow) → HIP (green arrow). Electrode contact colors in A match the arrow colors in B. Cu, cuneus; HIP, hippocampus; PCC, posterior cingulate cortex; PCu, precuneus.

Pattern III (9 patients, cases 1, 4, 5, 7, and 15–19, 47.4%): Dorsal‐medial/default mode network (DMN)–limbic pathway. Activity propagates selectively along the dorsal stream to dorsal parietal and posterior midline regions, including SPL, Cu, PCu, PCC, and HIP (Figure 4).

**FIGURE 5:**
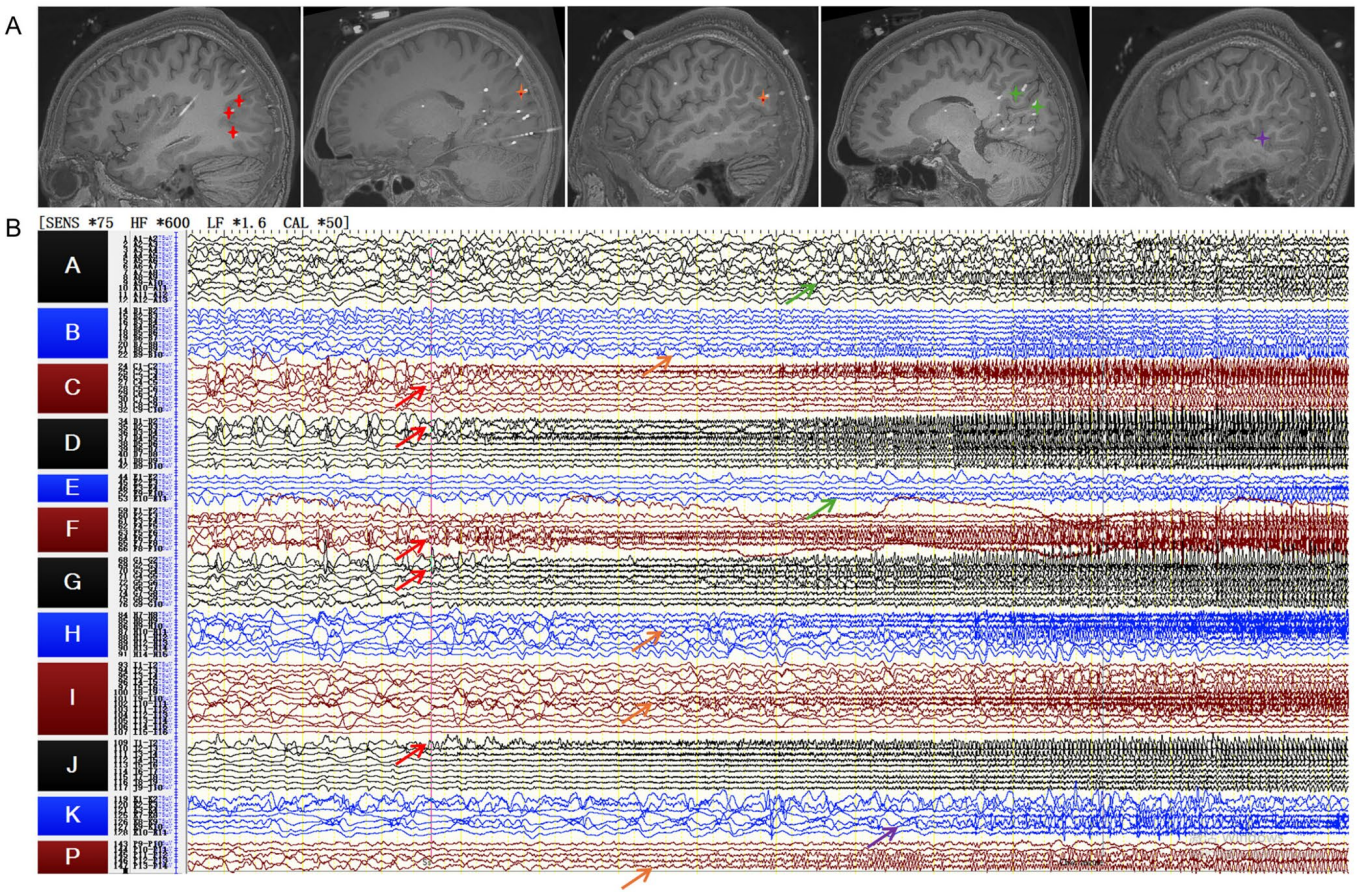
Case 8: SEEG electroclinical correlations. (A) Anatomical localization of key electrode contacts based on MRI/CT fusion imaging. (B) SEEG‐defined electro‐anatomical propagation pattern: MOG (red arrows) → TPOJ (orange arrow) → Cu and PCu (green arrow) → MTG (purple arrow). Electrode contact colors in A match the arrow colors in B. Cu, cuneus; MOG, middle occipital gyrus; MTG, middle temporal gyrus; PCu, precuneus; TPOJ, temporo‐parieto‐occipital junction.

Pattern IV (3 patients, cases 2, 8, and 9, 15.8%): Lateral occipital–TPOJ Pathway. Early involvement of the lateral occipital cortex and rapid propagation to the TPOJ, a key cortical integration hub, were consistently observed (Figure 5).

To further assess the robustness of these propagation‐pattern assignments, we applied the multimodal evaluation framework incorporating direct SEEG coverage, concordance across supportive electroclinical modalities, and case‐specific rationale (Table S1). Using this multimodal evaluation framework, 18 of 19 cases (94.7%) met the criteria for adequate direct SEEG coverage, whereas 1 of 19 cases (5.3%) was classified as having limited coverage. All 19 cases showed concordance with at least two of the three evaluative domains, and 11 of 19 cases (57.9%) showed concordance across all three. Notably, the single limited‐coverage case was also supported by all three domains. These findings support the internal consistency of the final propagation‐pattern assignments.

#### 3.4.5 Phenotype—Seizure Onset Zone—Pathway

The synchronous analysis of symptomatology, the onset zone of epileptic seizures and SEEG propagation pattern identified these features, thereby enabling the recognition of the anatomical–electrophysiological–clinical phenotype (Figure 2).

Pattern I (restricted) corresponds to phenotype I (pure visual), confirming a highly localized network. Pattern II (ventral stream–limbic) is primarily linked to phenotype II (±visual‐automatism) but also contributes to phenotype III when motor networks are engaged. Pattern III (dorsal‐medial/DMN–limbic), the most common, underlies a broad symptom spectrum and connects to phenotypes II, III, IV, and V, highlighting its role as a central hub for both automatism and motor‐dominant seizures. Pattern IV (lateral occipital–TPOJ) is associated with phenotypes III and IV, indicating involvement in seizures featuring visual aura, oculomotor onset, prominent automatisms, or progression to motor activity.

### 3.5 Surgical Results

Based on the results of SEEG monitoring, combined with imaging and PET examinations, all patients underwent SEEG‐guided thermocoagulation ablation. Individual patient data on lesion location, SOZ, and surgical intervention (resection range or RFTC targets) are summarized schematically in Figure S1. The current average follow‐up period is 22.9 ± 12.8 months, with 12 cases at Engel Class I, four cases at Engel Class II, and three cases at Engel Class III. Seven patients underwent further epilepsy focus resection after thermocoagulation ablation; in this subgroup, six cases achieved Engel Class I and one case was at Engel Class II. Pathological examination revealed five cases of FCD, one case of gliosis, and one case of ganglioglioma.

## 4 Discussion

### 4.1 Study Overview

This study systematically analyzed 19 patients with drug‐resistant OLE based on SEEG. By integrating detailed seizure semiology with high‐resolution electrophysiological localization under the framework of the dual visual processing model, we identified five clinical phenotypes and characterized their relationships with four SEEG‐defined propagation patterns. The results showed that epileptic activity originating in the occipital lobe diffused along specific functional networks, including localized occipital loop (pattern I), ventral–limbic pathway (pattern II), dorsal‐medial/DMN–limbic pathway (pattern III), and lateral TPOJ pathway (pattern IV), thereby generating diverse clinical manifestations. The high temporal resolution of SEEG helped delineate these propagation pathways [8, 15], providing a mechanistic explanation for divergent clinical manifestations despite similar initial symptoms: differential engagement of key cortical hubs, particularly the TPOJ and PCC/PCu, may facilitate rapid cross‐network convergence and underlie broader clinical expression. These findings extend traditional symptom‐based descriptions and support a network‐based framework for understanding the pathophysiology of OLE.

### 4.2 Patient Characteristics and Noninvasive Evaluation

In our cohort, subtle MRI‐detected structural abnormalities were common, with FCD and gliosis as the most frequent etiologies, followed by encephalomalacia; one case involved a tumor. This aligns with literature reporting improved detection of subtle lesions due to advances in MRI quality and consequently fewer MRI‐negative cases [5]. Scalp EEG often showed diffuse or bilateral and rapidly spread to other lobes or the contralateral occipital lobe. Ictal discharges were more helpful for lateralization [16]. However, the deep location of the medial and inferior occipital surfaces makes initial electrical signals hard to detect on scalp EEG, and rapid discharge spread hinders clinical identification, often leading to misdiagnosis as extra‐occipital epilepsy and low diagnostic accuracy [2, 5, 17], especially for lesions of the cuneus near the parieto‐occipital sulcus, which more frequently yield contralateral scalp EEG abnormalities [18]. PET was useful for hypothesis generation and lesion localization, although hypometabolic regions were often extensive and nonspecific [19]. These noninvasive findings highlight the challenges of diagnosing occipital epilepsy and underscore the critical role of SEEG in identifying the epileptogenic zone.

### 4.3 Key Findings and Phenotype‐Propagation Pattern Correspondences Within the Dual‐Stream Architecture

These findings may help explain why OLE often mimics other epilepsies, especially in patients without visual auras. Most seizures rapidly involve both dorsal and ventral pathways, as well as limbic structures, leading to overlapping symptoms like visual auras, automatisms, and contralateral tonic posturing, making clinical localization difficult without SEEG [6, 20]. This network‐based perspective also helps explain why limited intracranial sampling may fail to capture early propagation outside the primary onset region.

Phenotype I (pure visual aura) corresponds to pattern I, characterized by localized propagation within occipital regions. In cases 3 and 10, seizure activity involved only short‐loop activation of the lesion, with no significant spread beyond the occipital cortex. The absence of objective motor or autonomic manifestations reflects a highly confined epileptogenic network limited to early visual and adjacent occipital areas. This pattern confirms that when propagation is minimal, OLE may present as isolated subjective visual symptoms, easily underrecognized or misattributed to non‐epileptic visual disturbances [21].

Phenotype II is associated with both pattern II and III, primarily involving the ventral stream–limbic pathway with early activation of the ventral visual stream. In cases 6 and 11–14, seizure activity showed selective propagation from occipital regions to the FuG/ITG, accompanied by consistent engagement of medial temporal limbic structures (PHG/HIP). This is closely related to the inferior longitudinal fasciculus (ILF) and lingual‐amygdala (Li‐AM) fiber bundle as reported in the literature [22, 23]. This ventral–limbic trajectory explains why automatisms dominate the clinical presentation despite occipital onset: once activity reaches ventral temporal and limbic circuits, the seizure semiology closely resembles that of temporal lobe epilepsy, often with absent, brief, or overshadowed visual auras [6, 24, 25]. Thus, the observed semiology is determined not solely by the onset zone but by the functional networks recruited during propagation, explaining why occipital‐onset seizures mimic temporal lobe seizures [26].

Phenotype III: (visual → ocular motor → evolving motor) emerged as the most network heterogeneous. Following a visual aura, the rapid progression to head‐eye deviation and contralateral tonic posturing reflects the swift propagation of the ictal discharge to dorsal parietal nodes and subsequently to premotor areas. This phenotype was associated with patterns II, III, and IV, indicating that motor evolution can arise via multiple pathways: ventral–limbic routes, dorsal parietal/DMN pathways, or through integrative hubs like the TPOJ. This heterogeneity is anatomically plausible given the occipital cortex's dense structural connectivity with parietal, temporal, and posteromedial regions, specifically short occipito‐parietal association fibers, the inferior fronto‐occipital fasciculus, the cingulum bundle, and the inferior longitudinal fasciculus, and the vertical occipital fasciculus, as demonstrated in prior tractographic studies [13, 22, 23, 27]. Pattern III was the most common in our cohort and involved preferential propagation along the dorsal stream to parietal and posterior midline regions (SPL/IPS/PCu/PCC), followed by limbic engagement (including HIP where sampled). Such propagation may produce oculomotor signs, head deviation, and asymmetric tonic posturing, suggesting rapid access to motor planning and execution systems and aligning with the dorsal stream's role in spatial awareness and visually guided action, which explains frontal‐ or parietal‐like motor symptoms in OLE [28, 29]. More broadly, this phenotype illustrates that similar motor output via different initial pathways highlights the convergence of multiple streams onto motor planning and execution networks.

Phenotype IV (oculomotor onset → evolving motor phenotype) is characterized by seizures beginning with objective oculomotor signs and no clear visual aura, primarily linked to pattern IV and less commonly to pattern III. This suggests involvement of extra‐striate visual cortex with limited role in conscious vision, while early oculomotor signs indicate rapid spread to cortical eye movement areas within the dorsal stream and its junctions [30]. The TPOJ likely acts as an important multimodal hub that facilitates rapid cross‐network propagation and shifting early semiology toward objective signs, potentially masking subjective visual symptoms [31]. As a result, this hub‐mediated pathway produces semiology that appears extra‐occipital early in the seizure despite an occipital origin.

Phenotype V (behavioral arrest onset → oculomotor/motor) may reflect particularly early disturbance of dorsal‐medial/DMN–limbic spread. Seizures spread from the occipital region (Cu/SOG/MOG) through posterior midline structures (PCu/PCC), then to cingulate and medial premotor areas before motor generalization. The PCu and PCC are key posterior hubs of the DMN, which supports self‐referential processing, awareness, and behavioral responsiveness [32, 33]. Their early ictal involvement likely contributes to the initial behavioral arrest in this phenotype. This is consistent with prior evidence linking DMN disruption to impaired consciousness and behavioral arrest in both temporal and extratemporal epilepsies [32, 34]. In our cohort, the close association between Phenotype V and Pattern III, which prominently involves the PCC/PCu, supports this mechanism. These posterior midline regions may therefore act both as relay points for dorsal‐medial seizure spread and as functionally significant nodes whose early disruption underlies the characteristic semiology [34].

Overall, OLE's clinical expression depends more on early propagation networks than on occipital onset itself. Seizure propagation in posterior cortex epilepsy is not limited to a single anatomical pathway [22, 35]. Our classification was intended to identify the predominant electroclinical propagation pattern rather than an exclusive pathway. Cross‐stream propagation may occur, especially during later seizure evolution. Cases were classified into four patterns based on the predominant early electroclinical trajectory, which was defined by dominant engagement of downstream key nodes on SEEG and supported by concordant electroclinical findings. This framework recognizes the network‐based, potentially overlapping nature of seizure spread while maintaining clinically meaningful distinctions among the four principal patterns.

In interpreting these phenotype–propagation correspondences, it is important to consider potential effects of ASM tapering during SEEG monitoring, which may introduce electroclinical variability by lowering seizure threshold or amplifying features like propagation speed or GTCS likelihood [36, 37]. In our cohort, tapering was implemented conservatively to increase seizure yield, and existing evidence suggests it typically enhances detection of intrinsic epileptic networks without fundamentally altering their organization [36, 38]. We therefore analyzed only seizures consistent with habitual semiology, excluding atypical post‐tapering events. Thus, the reported associations reflect representative habitual seizures.

### 4.4 Clinical Translation: Network Dynamics Informing SEEG Strategy and Surgical Planning

Our analysis reveals several key principles of OLE network dynamics.

#### 4.4.1 Symptom Localization Value

The initial symptom is a reliable indicator of the SOZ, while subsequent symptoms are direct reflections of the propagation pathway engaged.

Epilepsy network dynamics exhibit selective, nonrandom propagation along preexisting functional pathways. This pathway selectivity is consistent with findings in occipital epilepsy, where semiology reflects stable electro‐anatomical networks rather than isolated foci. Yet, distinct preferential pathways can be dynamically recruited across seizures from the same epileptogenic zone, leading to divergent clinical phenotypes within individuals, demonstrating that seizure semiology is a state‐dependent output of a flexible network. This further suggests that phenotypic variability does not necessarily indicate a different anatomical route, but may instead arise from quantitative differences in seizure dynamics, including propagation speed, synchronization, intensity, duration, and subtle variation in onset location within a functionally heterogeneous epileptogenic zone [35, 39, 40]. Accordingly, our classification framework is grounded in the dominant early electroclinical propagation pattern most relevant to symptom generation, rather than later nonspecific spread or secondary generalization.

#### 4.4.2 Key Hubs Act as Accelerators and Switches

Early engagement of TPOJ and PCC/PCu drives widespread network synchronization, leading to early impaired consciousness and secondary generalization. In pattern IV, rapid TPOJ engagement enables multimodal integration, accounting for early oculomotor and automotor signs. In pattern III, PCC/PCu involvement drives DMN‐mediated synchronization, leading to similar network‐level effects. Although intracranial sampling limits definitive conclusions about hub necessity, consistent early engagement supports their role as switches that facilitate the transition from focal propagation to distributed epileptic networks.

#### 4.4.3 Clinical Implications for Implantation Strategy and Surgical Planning

Our findings suggest a phenotype‐guided SEEG approach may be useful for presurgical hypothesis generation. Preoperative identification of the dominant clinical phenotype may help guide electrode placement to the suspected propagation network, such as the ventral stream–limbic versus dorsal hub‐integrated pathways, shifting from mere anatomical localization to functional network mapping. In addition, white matter connectivity–based parcellation may refine understanding of occipital functional organization and improve clinical–anatomical correlation interpretation [41]. Together, these approaches may help delineate patient‐specific epileptogenic networks and optimize surgical targeting.

### 4.5 Surgical Treatment and Postoperative Pathology

In this cohort, most patients achieved Engel I after SEEG‐guided resection or thermal ablation, suggesting the effectiveness of these procedures for occipital lobe epilepsy when the epileptogenic zone is fully treated. Focal cortical dysplasia and gliosis were the main pathologies, suggesting favorable outcomes when the epileptogenic network is adequately treated. The high seizure‐free rate likely reflects alignment among seizure semiology, propagation network mapping, and surgical planning, underscoring the value of a network‐based classification system in preoperative decision making [42, 43]. For patients with multifocal or dual‐network involvement, comprehensive SEEG sampling and broader or multi‐lobar disconnection strategies may be required to prevent recurrence from incomplete network disconnection.

### 4.6 Limitations of This Study

This study is limited by its single‐center, retrospective design and small sample size, which reduce statistical power for subgroup analyses. Seizure propagation was evaluated through visual SEEG inspection without quantitative connectivity metrics. Electrode sampling was hypothesis‐driven, potentially biasing toward presurgically suspected regions—especially deep midline structures like the PCC/PCu and limbic areas. Subjective auras may be underreported due to early consciousness impairment from rapid seizure spread, which could influence phenotypic classification. Formal false positive/negative analysis was not feasible, given the absence of a gold standard for such classifications. While atypical post‐tapering events were excluded, subtle effects of ASM withdrawal on network excitability, propagation, or semiological expression cannot be ruled out. Accordingly, the phenotype–propagation associations remain exploratory and hypothesis‐generating, necessitating prospective multicenter validation with standardized network sampling and multimodal quantitative analyses.

## 5 Conclusion

In conclusion, this study proposes a preliminary anatomical–electrophysiological–clinical classification for OLE, suggesting that seizure semiology may be shaped by dynamic, pathway‐specific propagation of epileptic activity along preexisting functional visual and association networks. A phenotype‐oriented SEEG approach offers initial insights into occipital seizure network organization and may inform presurgical evaluation and individualized surgical planning.

## Funding

This work was funded by the Youth Fund of the Health Commission of Huangpu District, Shanghai (HLQ202203); the Outstanding Youth Talent Training Program of Ruijin Hospital Luwan Branch, Shanghai Jiao Tong University School of Medicine (YQ202313).

## Ethics Statement

This study was approved by the Ruijin Hospital Luwan Branch Ethics Committee, Shanghai JiaoTong University School of Medicine (approval number: LWEC2024017). All patients provided written informed consent prior to surgery.

## Conflicts of Interest

The authors declare no conflicts of interest.

## Supporting information


**Figure S1:** Patient‐specific schematic maps of lesion location, seizure onset zone (SOZ), radiofrequency thermocoagulation (RFTC) targets, and surgical resection extent. Each patient is presented in a single panel showing lateral and medial views of the involved hemisphere. Red indicates the lesion location, defined as the structural abnormality on preoperative MRI or, in MRI‐negative cases, the predominant hypometabolic region on FDG‐PET. Green dots indicate the SOZ, corresponding to electrode contacts at which ictal onset was identified on SEEG recordings. Purple circles show RFTC targets guided by SEEG. All 19 patients received RFTC. Resection (blue) is shown only for the seven who underwent curative surgery; the other 12 had RFTC ablation only.
**Table S1:** SEEG electrode coverage verification, supportive multimodal evidence, and rationale for final propagation‐pattern assignment.
**Table S2:** Impact of antiseizure medication reduction on seizure characteristics during SEEG monitoring.

## Data Availability

The data that support the findings of this study are available from the corresponding author upon reasonable request.

## References

[1] J. E. Adcock and C. P. Panayiotopoulos , “Occipital Lobe Seizures and Epilepsies,” Journal of Clinical Neurophysiology 29, no. 5 (2012): 397–407, 10.1097/WNP.0b013e31826c98fe. 23027097

[2] V. Salanova , F. Andermann , A. Oliver , T. Rasmussen , and L. F. Quesney , “Occipital Lobe Epilepsy: Electroclinical Manifestations, Electrocorticography, Cortical Stimulation and Outcome in 42 Patients Treated Between 1930 and 1991: Surgery of Occipital Lobe Epilepsy,” Brain 115, no. 6 (1992): 1655–1680, 10.1093/brain/115.6.1655. 1486456

[3] W. T. Blume , S. Wiebe , and L. M. Tapsell , “Occipital Epilepsy: Lateral Versus Mesial,” Brain 128, no. 5 (2005): 1209–1225, 10.1093/brain/awh45. 15788545

[4] S. Kun Lee , S. Young Lee , D. W. Kim , D. Soo Lee , and C. K. Chung , “Occipital Lobe Epilepsy: Clinical Characteristics, Surgical Outcome, and Role of Diagnostic Modalities,” Epilepsia 46, no. 5 (2005): 688–695, 10.1111/j.1528-1167.2005.56604.x. 15857434

[5] K. Yilmaz and E. Y. Karatoprak , “Epilepsy Classification and Additional Definitions in Occipital Lobe Epilepsy,” Epileptic Disorders 17, no. 3 (2015): 299–307, 10.1684/epd.2015.0767. 26299344

[6] A. Marchi , F. Bonini , S. Lagarde , et al., “Occipital and Occipital ‘Plus’ Epilepsies: A Study of Involved Epileptogenic Networks Through SEEG Quantification,” Epilepsy & Behavior 62 (2016): 104–114, 10.1016/j.yebeh.2016.06.014. 27454330

[7] H. Angus‐Leppan and T. A. Clay , “Adult Occipital Lobe Epilepsy: 12‐Years on,” Journal of Neurology 268, no. 10 (2021): 3926–3934, 10.1007/s00415-021-10557-y. 33900448

[8] D. Samanta , “Recent Developments in Stereo Electroencephalography Monitoring for Epilepsy Surgery,” Epilepsy & Behavior 135 (2022): 108914, 10.1016/j.yebeh.2022.108914. 36116362

[9] M. A. Goodale and A. D. Milner , “Two Visual Pathways—Where Have They Taken Us and Where Will They Lead in Future?,” Cortex 98 (2018): 283–292, 10.1016/j.cortex.2017.12.002. 29288012

[10] G. Rizzolatti and M. Matelli , “Two Different Streams Form the Dorsal Visual System: Anatomy and Functions,” Experimental Brain Research 153, no. 2 (2003): 146–157, 10.1007/s00221-003-1588-0. 14610633

[11] S. Fagioli and E. Macaluso , “Attending to Multiple Visual Streams: Interactions Between Location‐Based and Category‐Based Attentional Selection,” Journal of Cognitive Neuroscience 21, no. 8 (2009): 1628–1641, 10.1162/jocn.2009.21116. 18823252

[12] C. Koutsarnakis , S. Komaitis , E. Drosos , et al., “Mapping the Superficial Morphology of the Occipital Lobe: Proposal of a Universal Nomenclature for Clinical and Anatomical Use,” Neurosurgical Review 44, no. 1 (2019): 335–350, 10.1007/s10143-019-01212-2. 31758336

[13] C. M. Baker , J. D. Burks , R. G. Briggs , et al., “A Connectomic Atlas of the Human Cerebrum—Chapter 9: The Occipital Lobe,” Operative Neurosurgery 15, no. S1 (2018): S372–S406, 10.1093/ons/opy263. 30260435 PMC6888039

[14] P. Perucca , F. Dubeau , and J. Gotman , “Intracranial Electroencephalographic Seizure‐Onset Patterns: Effect of Underlying Pathology,” Brain 137, no. pt. 1 (2014): 183–196, 10.1093/brain/awt299. 24176980

[15] F. Bartolomei , S. Lagarde , F. Wendling , et al., “Defining Epileptogenic Networks: Contribution of SEEG and Signal Analysis,” Epilepsia 58, no. 7 (2017): 1131–1147, 10.1111/epi.13791. 28543030

[16] M. Cheval , M. Ferrand , S. Colnat‐Coubois , et al., “Patterns of Ictal Surface EEG in Occipital Seizures: A Simultaneous Scalp and Intracerebral Recording Study,” Clinical Neurophysiology 168 (2024): 83–94, 10.1016/j.clinph.2024.10.012. 39481134

[17] B. I. Ludwig and C. A. Marsan , “Clinical Ictal Patterns in Epileptic Patients With Occipital Electroencephalographic Foci,” Neurology 25, no. 5 (1975): 463–471, 10.1212/wnl.25.5.463. 1169704

[18] O. Vilela‐Filho , H. F. Silva‐Filho , L. C. Goulart , P. C. Ragazzo , and F. M. Arruda , “A New Strategy for Treating Drug‐Resistant Focal Aware Seizures: Thalamic Specific Nuclei Deep Brain Stimulation. Illustrative Case,” Journal of Neurosurgery: Case Lessons 6, no. 8 (2023): CASE23303, 10.3171/CASE23303. 37728299 PMC10555561

[19] J. Zhuang , L. Fei , H. Li , et al., “Anatomical‐Electroclinical Phenotypes and SEEG‐Defined Network Patterns in Pure Insular Lobe Epilepsy: A Study of 20 Cases,” Epilepsy Research 215 (2025): 107558, 10.1016/j.eplepsyres.2025.107558. 40328020

[20] B. C. Jobst , P. D. Williamson , V. M. Thadani , et al., “Intractable Occipital Lobe Epilepsy: Clinical Characteristics and Surgical Treatment,” Epilepsia 51, no. 11 (2010): 2334–2337, 10.1111/j.1528-1167.2010.02673.x. 20662891

[21] R. Mahmud and H. Sina , “Presentation, Etiology, Outcome, and Differentiation of Visual Semiology of Adult Occipital Epilepsy From Visual Aura of Migraine Headache: A Prospective Study in a Tertiary Care Center in Bangladesh,” Cureus 14, no. 4 (2022): e24186, 10.7759/cureus.24186. 35592185 PMC9109705

[22] F. Latini , M. Hjortberg , H. Aldskogius , and M. Ryttlefors , “The Classical Pathways of Occipital Lobe Epileptic Propagation Revised in the Light of White Matter Dissection,” Behavioural Neurology 2015 (2015): 1–10, 10.1155/2015/872645. PMC4430656 26063964

[23] A. H. Palejwala , K. P. O'Connor , P. Pelargos , et al., “Anatomy and White Matter Connections of the Lateral Occipital Cortex,” Surgical and Radiologic Anatomy 42, no. 3 (2019): 315–328, 10.1007/s00276-019-02371-z. 31734739

[24] J. V. Haxby , M. Mishkin , R. E. Carson , P. Herscovitch , M. B. Schapiro , and S. I. Rapoport , “Dissociation of Object and Spatial Visual Processing Pathways in Human Extrastriate Cortex,” Proceedings of the National Academy of Sciences of the United States of America 88, no. 5 (1991): 1621–1625, 10.1073/pnas.88.5.1621. 2000370 PMC51076

[25] L. Hu , F. Ding , S. Wang , and S. Wang , “MRI‐Negative Occipital Lobe Epilepsy Presenting as Gelastic Seizures,” Neurology India 69, no. 6 (2021): 1813–1816, 10.4103/0028-3886.333525. 34979696

[26] A. Mcgonigal , F. Bartolomei , and P. Chauvel , “On Seizure Semiology,” Epilepsia 62, no. 9 (2021): 2019–2035, 10.1111/epi.16994. 34247399

[27] E. J. Bubb , C. Metzler‐Baddeley , and J. P. Aggleton , “The Cingulum Bundle: Anatomy, Function, and Dysfunction,” Neuroscience and Biobehavioral Reviews 92 (2018): 104–127, 10.1016/j.neubiorev.2018.05.008. 29753752 PMC6090091

[28] J. Jacobs , “Networks in Posterior Cortex Epilepsies,” Neurosurgery Clinics of North America 31, no. 3 (2020): 325–334, 10.1016/j.nec.2020.03.002. 32475483

[29] Y. Li , F. Kong , M. Ji , Y. Luo , J. Lan , and X. You , “Shared and Distinct Neural Bases of Large‐ and Small‐Scale Spatial Ability: A Coordinate‐Based Activation Likelihood Estimation Meta‐Analysis,” Frontiers in Neuroscience 12 (2019): 1021, 10.3389/fnins.2018.01021. 30686987 PMC6335367

[30] L. Maillard , M. Ferrand , O. Aron , et al., “Visual Phenomena and Anatomo‐Electro‐Clinical Correlations in Occipital Lobe Seizures,” Revue Neurologique (Paris) 178, no. 7 (2022): 644–648, 10.1016/j.neurol.2022.06.001. 35906139

[31] A. De Benedictis , H. Duffau , B. Paradiso , et al., “Anatomo‐Functional Study of the Temporo‐Parieto‐Occipital Region: Dissection, Tractographic and Brain Mapping Evidence From a Neurosurgical Perspective,” Journal of Anatomy 225, no. 2 (2014): 132–151, 10.1111/joa.12204. 24975421 PMC4111924

[32] A. Vanhaudenhuyse , Q. Noirhomme , L. J. Tshibanda , et al., “Default Networkconnectivity Reflects the Level of Consciousness in Non‐Communicative Brain‐Damaged Patients,” Brain 133, no. pt. 1 (2010): 161–171, 10.1093/brain/awp313. 20034928 PMC2801329

[33] R. L. Buckner , J. R. Andrews‐Hanna , and D. L. Schacter , “The Brain's Default Network: Anatomy, Function, and Relevance to Disease,” Annals of the New York Academy of Sciences 1124 (2008): 1–38, 10.1196/annals.1440.011. 18400922

[34] F. Fahoum , R. Zelmann , L. Tyvaert , et al., “Epileptic Discharges Affect the Default Mode Network—FMRI and Intracerebral EEG Evidence,” PLoS One 8, no. 6 (2013): e68038, 10.1371/journal.pone.0068038. 23840805 PMC3695970

[35] A. McGonigal , “Semiology and Epileptic Networks,” Neurosurgery Clinics of North America 31, no. 3 (2020): 373–385, 10.1016/j.nec.2020.03.003. 32475486

[36] D. Zhou , Y. Wang , P. Hopp , et al., “Influence on Ictal Seizure Semiology of Rapid Withdrawal of Carbamazepine and Valproate in Monotherapy,” Epilepsia 43, no. 4 (2002): 386–393, 10.1046/j.1528-1157.2002.45201.x. 11952768

[37] H. P. Zaveri , S. M. Pincus , I. I. Goncharova , et al., “A Decrease in EEG Energy Accompanies Anti‐Epileptic Drug Taper During Intracranial Monitoring,” Epilepsy Research 86, no. 2–3 (2009): 153–162, 10.1016/j.eplepsyres.2009.06.002. 19632096

[38] G. Ren , S. Hannan , K. Schiller , et al., “Impact of Antiseizure Medication Taper on Electroencephalographic Dynamics in Focal Epilepsy: A Stereoelectroencephalographic Study,” Epilepsia 67 (2026): 2585–2600, 10.1002/epi.70149. 41697244

[39] T. Proix , V. K. Jirsa , F. Bartolomei , M. Guye , and W. Truccolo , “Predicting the Spatiotemporal Diversity of Seizure Propagation and Termination in Human Focal Epilepsy,” Nature Communications 9, no. 1 (2018): 1088, 10.1038/s41467-018-02973-y. PMC5852068 29540685

[40] G. M. Schroeder , F. A. Chowdhury , M. J. Cook , et al., “Multiple Mechanisms Shape the Relationship Between Pathway and Duration of Focal Seizures,” Brain Communications 4, no. 4 (2022): fcac173, 10.1093/braincomms/fcac173. 35855481 PMC9280328

[41] M. Thiebaut De Schotten , M. Urbanski , R. Valabregue , et al., “Subdivision of the Occipital Lobes: An Anatomical and Functional MRI Connectivity Study,” Cortex 56 (2014): 121–137, 10.1016/j.cortex.2012.12.007. 23312799

[42] H. Kreinter , P. H. Espino , S. Mejía , et al., “Disrupting the Epileptogenic Network With Stereoelectroencephalography‐Guided Radiofrequency Thermocoagulation,” Epilepsia 65, no. 7 (2024): e113–e118, 10.1111/epi.18005. 38738924

[43] J. Shen , H. Tan , B. Wu , et al., “The SEEG Brain Network Predicts Epileptic Surgical Outcomes of Radiofrequency Thermocoagulation,” Epilepsy Research 219 (2026): 107715, 10.1016/j.eplepsyres.2025.107715. 41330179

