# Supplementary material for: Mapping the Phenotypic Spectrum of Occipital Lobe Epilepsy: A SEEG‐Defined Network Classification
Source: CNS Neurosci Ther. 2026 Aug 7;32(8):e71027. doi: 10.1002/cns.71027 (PMC13449936; doi:10.1002/cns.71027)
Supplement: Supplementary file 1 [file CNS-32-e71027-s001.docx]

Supplementary Table S1. SEEG electrode coverage verification, supportive multimodal evidence, and rationale for final propagation pattern assignment

| Pat. | Ventral stream–limbic key nodes | | Dorsal-medial/DMN–limbic key nodes | | TPOJ | Predominant propagation pattern | Coverage adequacy | Supportive multimodal evidence for pathway inference | Rationale for final pattern assignment |
| --- | --- | --- | --- | --- | --- | --- | --- | --- | --- |
|  | (FuG, ITG) | (PHG, HIP) | (SPL, IPS, PCu) | (PCC, PHG, HIP) |  |  |  |  |  |
| 1 | F | F | F | F | N | III | Adequate | Validated (semiology + EEG + PET) | Semiology: Sz1 motor evolution, Sz2 automatisms;  Scalp EEG: Posterior → central/temporal propagation.  PET: L Cu/POS/PCu/HIP hypometabolism;  Coverage: Cu/ PCu/POS/PCC/HIP fully covered. |
| 2 | F | N | F | F | F | IV | Adequate | Validated (EEG + PET) | Scalp EEG: R lateral occipital → temporal/ parietal propagation.  PET: R TPOJ/lateral occipital hypometabolism;  Coverage: TPOJ/lateral occipital fully covered. |
| 3 | F | N | N | N | N | I | Adequate | Validated (semiology + PET) | Semiology: pure visual aura without progression;  PET: L LG/IOG isolated hypometabolism;  Coverage: LG/IOG fully covered; extra-occipital sampling not indicated. |
| 4 | F | F | F | F | N | III | Adequate | Validated (semiology + PET) | Semiology: Motor arrest → motor;  PET: L MOG/PCC/SOG, bilateral temporal/HIP hypometabolism;  Coverage: MOG/PCC/PCu/ SPL/HIP fully covered. |
| 5 | F | N | F | F | N | III | Adequate | Validated (semiology + EEG+ PET) | Semiology: Motor arrest → motor;  Scalp EEG: R posterior →temporal/parietal propagation;  PET: R Cu/T/HIP hypometabolism;  Coverage: SOG/Cu/SPL/PCC/ fully covered. |
| 6 | F | N | N | N | N | II | Limited | Validated (semiology + EEG + PET) | Semiology: Sz1 automatisms, Sz2 motor evolution  Scalp EEG: L posterior →temporal propagation.  PET: L LG/FuG/IOG/T/HIP/PHG hypometabolism; Coverage: LG/FuG/ITG fully covered. |
| 7 | N | N | F | F | P | III | Adequate | Validated (semiology + PET) | Semiology: Sz1 visual aura → automatisms, Sz2 motor evolution  PET: R>L occipital/HIP, R SPL/Cu hypometabolism;  Coverage: Cu/PCC/SPL fully covered. |
| 8 | P | F | F | F | F | IV | Adequate | Validated (semiology + EEG + PET) | Semiology: visual obscuration → oculomotor → tonic posturing, consistent with TPOJ-mediated rapid motor evolution;  PET: R MOG/SPL/HIP hypometabolism;  Scalp EEG: R occipital →temporal/parietal propagation.  Coverage: MOG/TPOJ/PCu/HIP sampled |
| 9 | F | F | F | F | F | IV | Adequate | Validated (semiology + EEG + PET) | Semiology: oculomotor onset → automatisms → tonic posturing;  Scalp EEG: L pos. → anterior propagation.  PET: L IOG/T/PHG/HIP hypometabolism;  Coverage: Lateral occipital /TPOJ/ fully covered |
| 10 | P | N | F | P | N | I | Adequate | Validated (semiology + PET) | Semiology: pure visual aura without progression;  PET: Bilateral Cu hypometabolism;  Coverage: LG/Cu fully covered; |
| 11 | F | F | P | F | P | II | Adequate | Validated (semiology + scalp EEG + PET) | Semiology: pure automatisms without motor signs;  Scalp EEG: L posterior →temporal propagation.  PET: Bilateral (R>L) posterior/temporal hypometabolism;  Coverage: IOG/ITG/FuG/HIP fully covered; dorsal nodes not required for Pattern II |
| 12 | F | F | F | F | F | II | Adequate | Validated (semiology + EEG + PET) | Semiology: visual loss → oculomotor → automatisms;  Scalp EEG: R posterior →temporal propagation.  PET: L occipital/T /FuG/HIP hypometabolism;  Coverage: IOG/ITG/MOG/HIP fully covered |
| 13 | F | F | F | F | F | II | Adequate | Validated (semiology + scalp EEG + PET) | Semiology: dizziness/visual → oculomotor → motor, ventral stream with rapid secondary generalization;  Scalp EEG: L posterior →temporal propagation.  PET: L occipital/temporal/FuG/HIP hypometabolism  Coverage: IOG/ITG/MOG/HIP fully covered. |
| 14 | F | F | F | F | F | II | Adequate | Validated (semiology + EEG + PET) | Semiology: visual obscuration → head/eye deviation → GTCS, ventral stream with rapid motor evolution;  Scalp EEG: L posterior →temporal/parietal propagation.  Coverage: LG/FuG/IOG/ITG/HIP fully covered |
| 15 | F | F | F | F | N | III | Adequate | Validated (semiology + EEG + PET) | Semiology: Sz1 automatisms, Sz2 secondary generalization, both via Pattern III;  Scalp EEG: R posterior → temporal/frontal/central propagation.  PET: L LG/Cu/FuG, bilateral (R>L) HIP hypometabolism;  Coverage: LG/Cu/HIP/PCu fully covered |
| 16 | F | F | F | F | F | III | Adequate | Validated (semiology+ PET) | Semiology: motor arrest → oculomotor → version.  PET: L MOG/IPG/STG/MTG/HIP hypometabolism;  Coverage: SOG/SPL/PCu/PCC/MCC/HIP fully covered |
| 17 | N | N | F | F | F | III | Adequate | Validated (semiology+ PET) | Semiology: visual → oculomotor →motor  PET: L Cu/PCu/SOG/IOG/SPL/PCC/T/HIP hypometabolism;  Coverage: LG/PCu/SPL/PCC/HIP fully covered |
| 18 | F | F | F | F | F | III | Adequate | Validated (semiology+ PET) | Semiology: oculomotor onset → automatisms → version → GTCS;  PET: R Cu/PCu/SPL hypometabolism;  Coverage: Cu/PCu/PCC/HIP fully covered |
| 19 | F | F | F | F | F | III | Adequate | Validated (semiology + EEG + PET) | Semiology: visual → oculomotor →GTCS;  scalp EEG: R posterior → frontal/central propagation.  PET: R Cu/SOG/MOG, bilateral HIP hypometabolism;  Coverage: MOG/PCu/PCC/HIP/MTG fully covered |

This table summarizes SEEG coverage of key nodes used for pathway assignment, including multimodal supportive evidence for cases with partial or no direct sampling. Electrode placement was hypothesis-driven and individualized based on Phase I hypotheses, targeting suspected propagation networks rather than uniform sampling.

**Coverage status (F/P/N):**

Fully covered (F): ≥ 2 electrode contact confirmed in the ROI’s gray matter via post-implantation CT–MRI fusion.

Partially covered (P): Only one gray-matter contact fell within the ROI, or sampling was adjacent or suboptimal—making ROI involvement inferable only indirectly.

Not covered (N): No electrode contact sampled of the ROI.

**Definition of pattern-level coverage status:**

Adequate: the minimum defining key nodes required for assignment of the final propagation pattern were directly sampled.

Limited: one or more defining key nodes were incompletely sampled, but the final assignment was retained because supportive multimodal findings were concordant.

**Supportive multimodal evidence for pathway inference:**

(1) sequential progression of seizure semiology;

(2) temporal evolution on scalp EEG; and

(3) spatial distribution of FDG-PET hypometabolism.

For cases with incomplete direct sampling, pathway inference was considered supported when at least two of these three modalities were concordant with the SEEG-derived propagation pattern.

**Notes**: Pattern assignment was intended to identify the predominant electroclinical propagation pathway, rather than an exclusive or anatomically isolated stream. Cross-stream propagation may occur, particularly in later phases of seizure spread.

Abbreviations: FuG: fusiform gyrus; ITG: inferior temporal gyrus; PHG: parahippocampal gyrus; HIP: hippocampus; SPL: superior parietal lobule; IPS: intraparietal sulcus; PCu: precuneus; PCC: posterior cingulate cortex; DMN: default mode network; TPOJ: temporo-parieto-occipital junction; FDG-PET: fluorodeoxyglucose positron emission tomography.

Supplementary Table S2: Impact of antiseizure medication reduction on seizure characteristics during SEEG monitoring

| Pat. | Baseline AEDs (daily dose) | AED reduction details during SEEG | Seizures captured before reduction (n) | Seizures captured after reduction (n) | Concordance with habitual semiology / atypical seizures | Impact on final pattern classification |
| --- | --- | --- | --- | --- | --- | --- |
| 1 | OXC 1200mg  LCM 200mg | LCM 50% ×2 (day 6-7);  OXC 25% ×3 (day 8-10) | 0 | 2 | Concordant / none | No impact |
|  |  |  | 1 | 1 |  |  |
| 2 | LEV 1250mg  LCM 100mg | LCM 50% ×2 (day 6-7); LEV 33% ×3 (day 8-10) | 0 | 4 | Concordant / none | No impact |
| 3 | OXC 900mg | No taper | 15 | NA | Concordant / none | No impact |
| 4 | LTG 150mg  TPM 200mg | TPM 25% ×4 (day 6-9); LTG 33% ×2 (day 10-11) | 1 | 3 | Concordant / none | No impact |
| 5 | PHT 600mg  PB 90mg  CZP 8mg | PHT 33% ×3 (day 6-8); PB 33% ×3 (day 9-11); CZP not tapered | 2 | 4 | Concordant / none | No impact |
| 6 | OXC 1200mg  PER 8mg | OXC 25%×2 (day 6-7); PER not tapered | 6 | 13 | Concordant / none | No impact |
|  |  |  | 0 | 1 |  |  |
| 7 | CBZ 400mg  VPA 800mg | CBZ 50%×2 (day 6-7); VPA 25%×4 (day 8-11) | 4 | 11 | Largely concordant / 2 atypical seizures with contralateral version | No impact; atypical seizures were excluded from final classification |
|  |  |  | 1 | 5 |  |  |
| 8 | LTG 225mg  VPA 1250mg  TPM 200mg  PER 4mg | No taper | 22 | NA | Concordant / none | No impact |
| 9 | LEV 750mg | LEV 33% ×3 (day 6-8) | 2 | 4 | Concordant / none | No impact |
| 10 | VPA 1500mg | VPA 33% ×3 (day 6-8) | 1 | 3 | Concordant / none | No impact |
| 11 | VPA 2000mg  TPM 200mg  PER 4mg | TPM 50%×2 (day 6-7);  VPA 25%×2 (day 8-9); PER not tapered | 2 | 5 | Concordant / none | No impact |
| 12 | VPA 560mg  TPM 150mg | TPM 33% ×3 (day 6-8);  VPA not tapered | 6 | 12 | Concordant / none | No impact |
| 13 | LEV 1000mg | LEV 25%×4 (day 6-9) | 1 | 3 | Concordant / none |  |
| 14 | OXC 1500mg  LEV 1000mg  LCM 350mg | LEV 50%×2 (day 6-7);  OXC 25%×4 (day 8-11);  LCM 33%×1 (day12) | 2 | 3 | Largely concordant / 1 atypical seizure with contralateral version | No impact; atypical seizures were excluded from final classification |
| 15 | VPA 1000mg  LEV 1500mg | VPA 25%×2 (day 6-7); 50%×1 (day 8);  LEV 33%×2 (day 9-10) | 2 | 3 | Concordant / none | No impact |
|  |  |  | 1 | 4 |  |  |
| 16 | OXC 1200mg  VPA 800mg  CZP 2mg | OXC 25%×4 (day 6-9);  VPA 25%×2 (day 10-11);  CZP not tapered | 2 | 3 | Concordant / none | No impact |
| 17 | BRV 50mg  LCM 200mg  CLB 20mg | BRV 100% (day 6);  LCM 50%×2 (day 7-8)  CLB 50%×2 (day 9-10) | 1 | 4 | Concordant / none | No impact |
| 18 | UCPM | UCPM 100% (day 6) | 3 | 4 | Concordant / none | No impact |
| 19 | OXC 1500mg  PER 12mg | OXC 30%×3 (day 6-8);  PER 30%×2 (day 9-10) | 3 | 6 | Concordant / none | No impact |

This table summarizes antiseizure medication reduction during SEEG monitoring, including tapering details, changes in seizure frequency, semiology concordance with habitual events, and impact on propagation characteristics.

**Protocol overview:** Baseline ASMs were maintained during the initial 5-day post-implantation observation period whenever feasible. ASM tapering was initiated only when spontaneous seizure yield was absent or insufficient for electroclinical characterization, and was performed conservatively and individually. Final propagation pattern classification was based primarily on habitual seizures with concordant electroclinical features; atypical seizures post-substantial reduction were interpreted cautiously and excluded from primary classification.

**Impact assessment:** Impact on final pattern classification refers to whether ASM tapering altered the interpretation of the dominant early electroclinical propagation pattern relevant to pathway classification, rather than nonspecific later generalization.

**Abbreviations.** ASM: antiseizure medication; BRV: brivaracetam; CBZ: carbamazepine; CLB: clobazam; CZP: clonazepam; LCM: lacosamide; LEV: levetiracetam; LTG: lamotrigine; OXC: oxcarbazepine; PB: phenobarbital; PER: perampanel; PHT: phenytoin; TPM: topiramate; UCPM: unknown Chinese patent medicine; VPA: valproate.

Supplementary Figure 1. Patient-specific schematic maps of lesion location, seizure onset zone (SOZ), radiofrequency thermocoagulation (RFTC) targets, and surgical resection extent. Each patient is presented in a single panel showing lateral and medial views of the involved hemisphere. Red indicates the lesion location, defined as the structural abnormality on preoperative MRI or, in MRI-negative cases, the predominant hypometabolic region on FDG-PET. Green dots indicate the SOZ, corresponding to electrode contacts at which ictal onset was identified on SEEG recordings. Purple circles show RFTC targets guided by SEEG. All 19 patients received RFTC. Resection (blue) is shown only for the 7 who underwent curative surgery; the other 12 had RFTC ablation only.
